# Non-Panel Men and the Medical Directory

**Published:** 1914-04-18

**Authors:** F. L. Nicholls

**Affiliations:** Fulbourn


*To the Editor of* The Hospital.

### Non-Panel Men and the Medical Directory.

*To the Editor of* The Hospital.

Sir,—In reading over the "Resolutions" of the "National Medical Union" in The Hospital for April 11, I see nothing included to give the public information as to who are not National Insurance men. Surely it is only just and fair both to the public and to non-panel members of the profession that there should be some means of distinguishing between them and Insurance doctors.

They have shown grit in their endeavour to uphold the honour of the profession, and have no doubt in many instances suffered financially, if not mentally, by the strain imposed upon them, and surely they are entitled to some distinction.

Would it not be possible to approach the editors of the Medical Directory and get them to insert the query in their annual circular for insertion in the Directory? If they did not see their way to do this, would it not be well for every non-panel man to fail to return the circular? He would then, I believe, have an asterisk placed against his name, and this would in a great measure, if all acted up to this principle, distinguish the non-panel members of the profession.—I am, Sir, yours truly,

Fulbourn, April 12, 1914. F. L. Nicholls.

